# Second primary driver-negative lung adenocarcinoma following breast cancer treatment: a case report

**DOI:** 10.11604/pamj.2026.53.71.50789

**Published:** 2026-02-09

**Authors:** Zineb Khadrouf, Khadija Khadiri, Hafida Benmessaoud, Amina Essalihi, Oumaima Bouchra, Majda Taoudi Benchekrou, Fatima Amarir, Abdallah Naya, Mehdi Karkouri

**Affiliations:** 1 Laboratory of Integrative Biology, Faculty of Sciences Ain Chock, Hassan II University of Casablanca, Casablanca, Morocco,; 2 Pathology Department, Ibn Rochd University Hospital, Casablanca, Morocco,; 3 Laboratory of Cellular and Molecular Pathology, Faculty of Medicine and Pharmacy, Hassan II University of Casablanca, Casablanca, Morocco

**Keywords:** Driver-negative lung adenocarcinoma, second primary cancer, post-therapeutic carcinogenesis, case report

## Abstract

We present the case of a 60-year-old non-smoking woman previously treated for luminal B human epidermal growth factor receptor 2 (HER2)-positive invasive breast carcinoma with surgery, AC60 chemotherapy, trastuzumab, breast radiotherapy, and hormone therapy at the Mohammed VI Oncology Center in Casablanca, Morocco. Four years after completing treatment, she presented with respiratory symptoms and was diagnosed with a well-differentiated, lepidic-type mucinous primary lung adenocarcinoma, staged IIIA (pT2bN1M0). Molecular analysis showed the absence of epidermal growth factor receptor (EGFR) mutations, anaplastic lymphoma kinase (ALK) and ROS1 rearrangements, rearranged during transfection (RET) fusions, and MET exon 14 skipping, with intermediate programmed death-ligand 1 (PD-L1) expression, assessed by tumor proportion score (TPS) of 10%. The patient received neoadjuvant vinorelbine-cisplatin chemotherapy followed by volumetric modulated arc therapy (VMAT) thoracic radiotherapy at 66 Gy, achieving clinical and radiological stabilization. This case highlights the occurrence of a second driver-negative primary lung adenocarcinoma in a non-smoker and underscores the importance of integrated histopathological, immunohisto chemical, and targeted molecular evaluation in distinguishing primary tumors from metastases, as well as the potential role of post-therapeutic carcinogenesis.

## Introduction

The occurrence of a second primary lung cancer in a non-smoking patient who has previously been treated for breast cancer raises significant biological questions. Driver-negative lung adenocarcinomas constitute a distinct subgroup whose pathogenesis is not well understood, especially when they develop following treatment involving anthracyclines, alkylating agents, and radiotherapy [1,2]. Distinguishing between breast metastasis and a second primary lung cancer is essential, and this is based on histological, immunohistochemical, and molecular profiling analysis [3]. The development of subsequent primary cancers may reflect shared host susceptibility and/or long-term consequences of prior anticancer treatments. This case study presents a driver-negative mucinous primary lung adenocarcinoma in a non-smoking woman who survived HER2-positive breast cancer, offering a valuable model for investigating the mechanisms of secondary carcinogenesis.

## Patient and observation

**Patient information:** the 60-year-old postmenopausal woman (height: 160 cm; weight: 89 kg; BMI: 34.76 kg/m^2^), who was a non-smoker, with controlled hypertension and no personal or family history of cancer, was initially diagnosed with left breast HER2-positive luminal B invasive carcinoma of no special type (NST). Four years later, she consulted for new respiratory symptoms, including mild hemoptysis and chest pain. Her medical history was otherwise unremarkable, with no known genetic predisposition and no relevant psychosocial factors. Previous oncologic interventions included breast-conserving surgery with axillary lymph node dissection, which revealed 3 positive lymph nodes out of 20, adjuvant anthracycline-based chemotherapy (AC60), paclitaxel plus trastuzumab, breast radiotherapy, and sequential hormone therapy, with no evidence of recurrence during follow-up.

**Clinical findings:** the clinical examination remained completely normal throughout the follow-up period following the initial treatment, which covered a period of approximately four years. Subsequently, the patient consulted regarding the onset of bronchial syndrome, presenting with mild hemoptysis and chest pain. Despite these symptoms, her general condition remained stable, with a performance status of 1 (PS=1).

**Timeline of current episode:** following diagnosis of luminal B HER2-positive invasive breast NST carcinoma in the left breast, the patient received comprehensive care, including breast-conserving surgery, followed by adjuvant chemotherapy using the AC60 protocol, then treatment with paclitaxel combined with trastuzumab, followed by maintenance therapy with trastuzumab in combination with tamoxifen, administered as adjuvant therapy due to HER2 overexpression. Breast radiotherapy was performed according to recommendations, supplemented by sequential hormone therapy with tamoxifen. Oncological follow-up was regular and without recurrence for a period of approximately four years after the initial diagnosis, until the discovery of a suspicious right mediobasal lesion on chest imaging, suggesting the appearance of a well-differentiated mucinous primary pulmonary adenocarcinoma with a predominant lepidic architecture.

**Figure 1:**
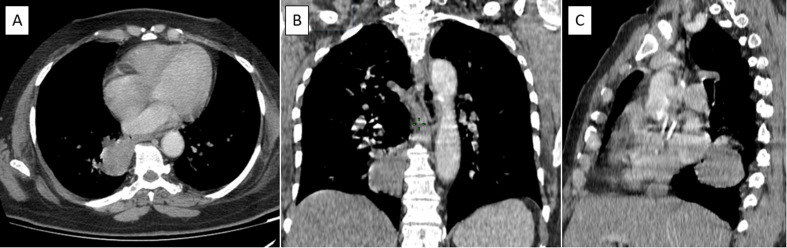
thoracic computed tomography demonstrating a right mediobasal pulmonary mass; axial (A); coronal (B); and sagittal (C) views

**Figure 2:**
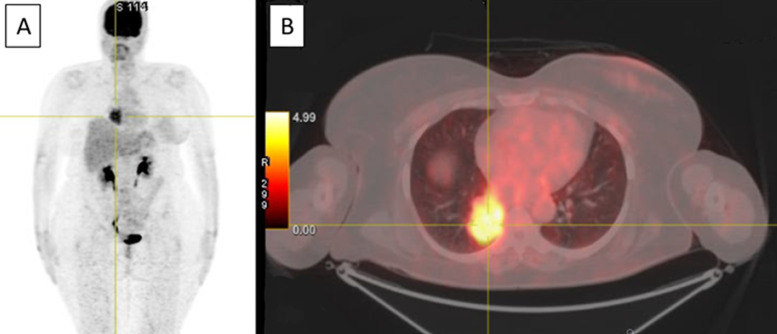
18F-fluorodeoxyglucose (18F-FDG) positron emission tomography-computed tomography (PET-CT) findings; A) full-body maximum intensity projection (MIP) image showing focal hypermetabolic uptake in the right lung; B) axial fused PET-CT image demonstrating hypermetabolic activity corresponding to the right mediobasal pulmonary lesion

**Figure 3:**
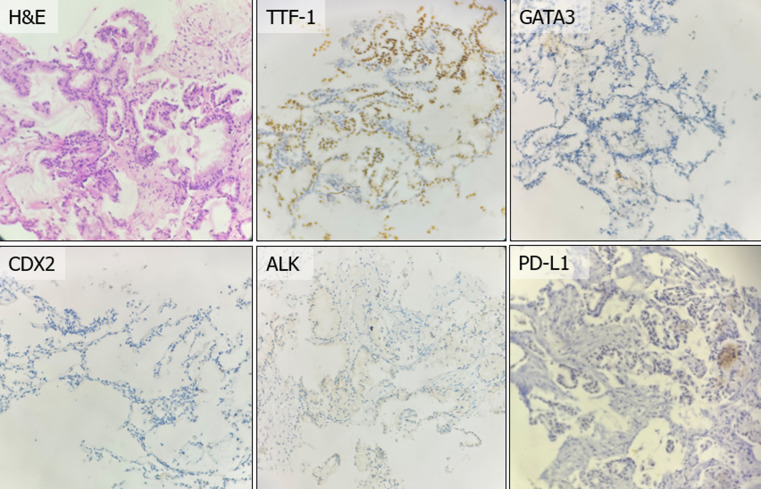
histopathological and immunohistochemical features supporting a primary invasive mucinous lung adenocarcinoma; hematoxylin and eosin (H&E) staining shows a mucinous adenocarcinoma with predominant lepidic growth; immunohistochemistry demonstrates TTF-1 positivity, GATA3 negativity, CDX2 negativity, ALK score 0, and PD-L1 tumor proportion score (TPS) 10% (original magnification x200)

**Figure 4:**
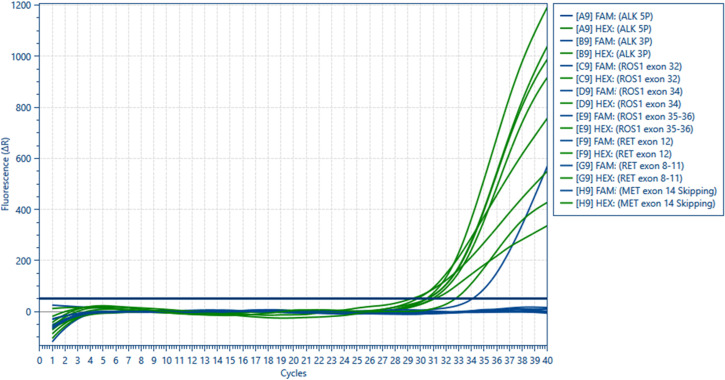
multiplex real-time reverse transcription-polymerase chain reaction (RT-PCR) analysis showing no detectable ALK, ROS1, or RET rearrangements and no MET exon 14 skipping

**Diagnostic assessment:** the diagnostic assessment included a combination of imaging, endoscopic evaluation, histopathology, and molecular testing. A thoracic computed tomography (CT) scan revealed a suspicious right mediobasal pulmonary process (Figure 1), which was further characterized by positron emission tomography-computed tomography (PET-CT) as a hypermetabolic lesion without distant metastases (Figure 2). Initial bronchoscopy was non-contributory, necessitating a percutaneous transthoracic biopsy (PBTP). Histopathological examination identified an invasive mucinous adenocarcinoma with a predominant lepidic architecture, positive for thyroid transcription factor-1 (TTF-1) and negative for GATA3 and CDX2. PD-L1 testing showed a TPS of 10%, and ALK immunohistochemistry was negative (score 0) (Figure 3). Targeted molecular testing was performed using qPCR for EGFR mutation analysis (Idylla™) and one-step RT-qPCR for the detection of ALK, ROS1, and RET rearrangements and MET exon 14 skipping (EasyPGX® ready ALK/ROS1/RET/MET) (Figure 4). No actionable alterations were identified among the tested targets. Diagnostic challenges included the initial non-diagnostic bronchoscopy and the absence of next-generation sequencing (NGS) access, limiting the depth of molecular characterization.

**Diagnosis:** the final diagnosis was a primary invasive mucinous adenocarcinoma of the lung, confirmed by morphology and immunohistochemistry, and molecularly classified as driver-negative. Alternative diagnoses considered included a metastasis from the prior HER2-positive breast carcinoma, which was ruled out based on the absence of breast markers (GATA3-), the presence of TTF-1 positivity, and the distinct mucinous phenotype. According to the AJCC 8th edition, the tumor was staged pT2bN1M0, corresponding to stage IIIA non-small-cell lung cancer (NSCLC). The prognosis reflects a locally advanced tumor without distant spread.

**Therapeutic interventions:** given the inoperable status of the lung tumor, chemotherapy with intravenous vinorelbine and cisplatin was administered. Thoracic radiotherapy (VMAT 66 Gy) was also delivered. Hormone therapy with anastrozole was continued in parallel for breast cancer, as a follow-up to tamoxifen.

**Follow-up and outcome of interventions:** reassessment with metabolic imaging and brain magnetic resonance imaging (MRI) showed no evidence of secondary progression. PET imaging confirmed metabolic stability of the pulmonary lesion, with no new lesions or extra-thoracic disease. The patient maintained a preserved general condition (performance status = 1), tolerated treatment well, and experienced no severe adverse events. She is currently under regular clinical and radiological surveillance.

**Patient perspective:** the patient reported understanding the distinction between a second primary lung cancer and metastatic breast disease. She tolerated treatment well and remains compliant with ongoing clinical and radiological follow-up.

**Informed consent:** the study protocol was conducted under the ethical principles, the ethical committee of Ibn Rochd University Hospital of Casablanca approved this study under the reference number Dossier No3/2022 (CHUIR-EC/03-2022), dated 13 September 2022. The case report is presented anonymously, and all identifying information has been removed to protect patient confidentiality. The patient has expressed her consent to the publication of this report.

## Discussion

This case illustrates the diagnostic and biological complexity associated with the development of a second primary lung adenocarcinoma in a non-smoking woman previously treated for HER2-positive breast carcinoma. Several strengths enhance the scientific value of this observation. The distinction between a primary pulmonary neoplasm and a breast metastasis was established through a robust combination of histological architecture and immunohistochemical profiling. The mucinous, predominantly lepidic architecture, together with the expression of TTF-1 and absence of GATA3, supported a primary pulmonary origin. The pulmonary lesion developed after a disease-free interval of approximately four years and arose in the contralateral lung relative to the irradiated breast, reinforcing the interpretation of an independent malignant process rather than locoregional spread. The targeted reverse transcription-polymerase chain reaction (RT-PCR) molecular analysis excluded common actionable driver alterations (EGFR mutations, ALK/ROS1/RET rearrangements, MET exon 14 skipping), allowing precise characterization of this tumor as driver-negative. The clinical course, multidisciplinary management, and radiological follow-up were thoroughly documented, enabling a rigorous evaluation of therapeutic response in a non-operable stage IIIA mucinous adenocarcinoma. Nevertheless, this case also presents several limitations that must be acknowledged. The main limitations include the absence of comprehensive genomic profiling by NGS, lack of germline genetic testing, and the inherently descriptive nature of a single-case observation, which precludes definitive causal inference regarding the mechanisms of secondary carcinogenesis.

Epidemiological studies have shown that breast carcinoma survivors have a moderately increased long-term risk of developing a second primary lung cancer, particularly after radiotherapy; however, this excess risk predominantly affects the ipsilateral lung relative to the irradiated breast, making a direct radio-induced mechanism less likely in cases of contralateral pulmonary tumors [4]. Systemic chemotherapy, especially anthracyclines and alkylating agents, has been shown to induce persistent DNA double-strand breaks, chromosomal instability, and long-lasting mutational damage, which may contribute to secondary carcinogenesis several years after exposure [5]. In parallel, large-scale pan-cancer genomic studies have identified specific mutational signatures associated with prior radiotherapy and chemotherapeutic agents, providing molecular evidence that anticancer treatments can leave durable genomic imprints predisposing to subsequent malignancies [6]. From a pathological standpoint, international WHO and IASLC recommendations emphasize the integration of histological features, immunohistochemistry, and molecular profiling to distinguish second primary lung cancers from breast metastases in cancer survivors, particularly in cases with mucinous differentiation [3,7]. Driver-negative lung adenocarcinomas, particularly those with mucinous histology, are recognized as a distinct biological subgroup with specific morphological and molecular features and are often reported among non-smokers, suggesting heterogeneous carcinogenic mechanisms beyond classical tobacco-related pathways [8].

From a scientific standpoint, the interpretation of this lesion as a second primary lung adenocarcinoma rather than metastatic breast recurrence is supported by converging arguments, including a distinct mucinous-lepidic morphology consistent with a pulmonary adenocarcinoma subtype, an immunophenotype favoring lung origin (TTF-1 positivity with absence of breast markers such as GATA3), and a four-year disease-free interval compatible with criteria for multiple primary malignancies. The absence of a disseminated pattern on PET-CT and brain MRI further supports the likelihood of an independent lung primary. Molecular analysis by RT-PCR excluded major actionable alterations, allowing classification as driver-negative within the limits of the tested targets, while acknowledging that additional mucinous-associated drivers would require NGS for exclusion. With respect to causality, a direct radiation-induced mechanism appears less likely given the contralateral tumor location, whereas prior systemic treatments, including anthracycline-based chemotherapy, taxanes, and trastuzumab, may have contributed through cumulative genotoxic stress and long-term genomic instability; an underlying host susceptibility cannot be excluded.

## Conclusion

This case highlights the possibility of driver-negative primary lung adenocarcinoma occurring in a non-smoking woman previously treated for HER2-positive breast cancer. It underscores the importance of integrated histological and targeted molecular analysis to distinguish multiple primary tumors from metastases and highlights the possible contribution of post-therapeutic carcinogenesis. This report underscores the need for comprehensive molecular characterization in patients presenting with new pulmonary lesions after prior cancer therapy, and the importance of long-term surveillance to detect secondary primary tumors at a potentially treatable stage.
